# Diet, Eating Habits, and Lifestyle Factors Associated with Adequate Sleep Duration in Children and Adolescents Living in 5 Mediterranean Countries: The DELICIOUS Project

**DOI:** 10.3390/nu17071242

**Published:** 2025-04-02

**Authors:** Justyna Godos, Alice Rosi, Francesca Scazzina, Maria Antonieta Touriz Bonifaz, Francesca Giampieri, Osama Abdelkarim, Achraf Ammar, Mohamed Aly, Evelyn Frias-Toral, Juancho Pons, Laura Vázquez-Araújo, Josep Alemany-Iturriaga, Lorenzo Monasta, Ana Mata, Adrián Chacón, Pablo Busó, Giuseppe Grosso

**Affiliations:** 1 Department of Biomedical and Biotechnological Sciences, University of Catania, 95123 Catania, Italy; justyna.godos@unict.it; 2 Center for Human Nutrition and Mediterranean Foods (NUTREA), University of Catania, 95123 Catania, Italy; 3 Human Nutrition Unit, Department of Food and Drug, University of Parma, 43124 Parma, Italy; 4 Facultad de Ciencias de la Salud, Universidad Católica de Santiago de Guayaquil, Av. Pdte. Carlos Julio Arosemena Tola, Guayaquil 090615, Ecuador; 5 Facultad de Ciencias Médicas, Universidad de Guayaquil, Avenida 10 NO, Guayaquil 090613, Ecuador; 6 Department of Clinical Sciences, Università Politecnica delle Marche, 60131 Ancona, Italy; 7 Research Group on Food, Nutritional Biochemistry and Health, Universidad Europea del Atlántico, Isabel Torres 21, 39011 Santander, Spain; 8 Joint Laboratory on Food Science, Nutrition, and Intelligent Processing of Foods, Polytechnic University of Marche, Italy, Universidad Europea del Atlántico Spain and Jiangsu University, China at Polytechnic University of Marche, 60130 Ancona, Italy; 9 International Research Center for Food Nutrition and Safety, Jiangsu University, Zhenjiang 212013, China; 10 Faculty of Sport Sciences, Assiut University, Assiut 71515, Egyptmohamed.aly@aun.edu.eg (M.A.); 11 Department of Training and Movement Science, Institute of Sport Science, Johannes Gutenberg-University Mainz, 55122 Mainz, Germany; 12 Research Laboratory, Molecular Bases of Human Pathology, LR19ES13, Faculty of Medicine, University of Sfax, Sfax 3029, Tunisia; 13 High Institute of Sport and Physical Education of Sfax, University of Sfax, Sfax 3000, Tunisia; 14 Escuela de Medicina, Universidad Espíritu Santo, Samborondón 0901952, Ecuador; 15 Division of Research, Texas State University, 601 University Dr, San Marcos, TX 78666, USA; 16 Editorial Luis Vives (EDELVIVES), Carretera de Madrid, 50012 Zaragoza, Spain; 17 BCC Innovation, Technology Center in Gastronomy, Basque Culinary Center, 20009 Donostia-San Sebastián, Spain; lvazquez@bculinary.com; 18 Basque Culinary Center, Faculty of Gastronomic Sciences, Mondragon Unibertsitatea, 20009 Donostia-San Sebastián, Spain; 19 Department of Health, Nutrition and Sport, Universidad Internacional Iberoamericana, Campeche 24560, Mexico; 20 Universidade Internacional do Cuanza, Cuito EN 250, Bié, Angola; 21 Institute for Maternal and Child Health–IRCCS Burlo Garofolo, 34137 Trieste, Italy; lorenzo.monasta@burlo.trieste.it; 22 Technological Institute for Children’s Products & Leisure AIJU, 03440 Alicante, Spain

**Keywords:** sleep, determinants, diet quality, dietary habits, lifestyle behaviors, children, adolescents, Mediterranean area

## Abstract

**Background/Objectives**: Sleep is a fundamental physiological function that plays a crucial role in maintaining health and well-being. The aim of this study was to assess dietary and lifestyle factors associated with adequate sleep duration in children and adolescents living in five Mediterranean countries. **Methods**: Parents of children and adolescents taking part in an initial survey for the DELICIOUS project were examined to assess their children’s dietary and eating habits (i.e., meal routines), as well as other lifestyle behaviors (i.e., physical activity levels, screen time, etc.) potentially associated with adequate sleep duration (defined as 8–10 h according to the National Sleep Foundation). The youth healthy eating index (Y-HEI) was used to assess the diet quality of children and adolescents. Multivariate logistic regression analyses were performed to calculate the odds ratios (ORs) and 95% confidence intervals (CIs), indicating the level of association between variables. **Results**: A total of 2011 individuals participated in the survey. The adolescents and children of younger parents reported being more likely to have inadequate sleep duration. Among eating behaviors, having breakfast (OR = 2.23, 95% CI: 1.62, 3.08) and eating at school (OR = 1.33, 95% CI: 1.01, 1.74) were associated with adequate sleep duration. In contrast, children eating alone, screen time, and eating outside of the home were less likely to have adequate sleep duration, although these findings were only significant in the unadjusted model. After adjusting for covariates, a better diet quality (OR = 1.63, 95% CI: 1.24, 2.16), including higher intake of fruits, meat, fish, and whole grains, was associated with adequate sleep duration. **Conclusions**: Adequate sleep duration seems to be highly influenced by factors related to individual lifestyles, family and school eating behaviors, as well as diet quality.

## 1. Introduction

For humans, sleep is a physiological aspect that is essential for maintaining health and well-being, linked to a decrease in the awareness of external stimuli and a halt in physical movement [1]. The quality of sleep is affected by various factors, including diet, physical activity, as well as genetic and environmental influences [2]. The regulation of sleep is largely driven by complex neurobiological mechanisms within the brain, which control when we fall asleep, the different stages of sleep we experience, and when we wake up [3]. These processes are fundamental to maintaining overall health, as sleep plays a crucial role in restoring the body and also in growth, development, learning, memory, synaptic efficiency, the regulation of behavior and emotions, the strengthening of the immune system, and the removal of neurotoxic substances [4]. The duration of sleep in healthy people usually diminishes with age, with 7 to 9 h of sleep required from childhood to adulthood [5]. Shorter sleep duration rates have been linked to reduced well-being and poorer cardiometabolic health outcomes, including type-2 diabetes, high blood pressure, metabolic syndrome, and coronary artery disease [6]. Additionally, those who sleep less face a greater risk of developing mental health issues (such as depression), while suffering from poor sleep quality might even be a prodromal syndrome for more serious neurodegenerative conditions (including dementia and Parkinson’s disease) [7]. This interplay between sleep and various health conditions underscores the importance of prioritizing good sleep hygiene and addressing sleep-related issues early on to foster better health outcomes and enhance the overall quality of life [8].

Over the past few decades, a decrease in sleep quality has been reported globally, associated with increasing rates of insomnia, frequent disturbances during the night, difficulty staying asleep, and extended periods of dreaming [9]. This growing concern over poor sleep quality is becoming a widespread issue, impacting various age groups. Insomnia in children results in decreased focus, which can negatively affect their ability to learn [10]. In particular, the lack of sleep in adolescents has been recognized as a serious health risk for both physical and mental well-being, leading to a range of issues such as weakened immune function, poor academic performance, increased susceptibility to stress and anxiety, and a higher risk of developing chronic conditions like obesity and heart disease later in life [11]. This growing concern highlights the importance of addressing sleep habits and ensuring that adolescents obtain the recommended amount of rest for their overall health [12].

There are many factors that influence the duration and quality of sleep in children and adolescents: alongside genetic influences, various environmental aspects also play a role in determining sleep requirements, like caffeine intake, early school schedules, ongoing health issues, sleep disorders with neurological origins (e.g., obstructive sleep apnea and restless legs syndrome), the stress of achieving academic success, engaging in extracurricular activities, and maintaining a busy social life [13]. This issue can be influenced by specific circumstances and habits, such as prolonged sedentary behaviors and a lack of physical activity (helping with body tiredness promoting sleep), excessive light exposure, screen time, and brain stimulating activities, and the absence of a pre-sleep routine or stimulus may cause the child to struggle with falling asleep [14]. Recently, dietary habits have also been considered to potentially influence mental health and sleep quality [15]. Diet has been hypothesized to influence diverse sleep features [16], in both adults and younger generations, who have been reported to abandon traditional dietary patterns and adopt more Westernized diets [17,18]. Such trends are suggested to be part of an overall lifestyle shift toward unhealthy behaviors, which in turn might negatively affect sleep in younger generations [19]. Thus, addressing nutritional status among the young population and implementing tailored and effective strategies to increase adherence to a balanced diet to improve health status is of primary importance [20].

As it is important to investigate the overall lifestyle to identify factors potentially playing a role in sleep physiology in children and adolescents, this study aimed to identify the lifestyle and nutritional factors linked to sleep duration in younger populations across the Mediterranean region.

## 2. Materials and Methods

### 2.1. Study Design and Population

The current research is grounded in a cross-sectional analysis within the framework of the European-funded DELICIOUS project (UnDErstanding consumer food choices & promotion of healthy and sustainable Mediterranean Diet and LIfestyle in Children and adolescents through behavIOUral change actionS) [21]. For this study, a consumer survey was conducted among parents of children and adolescents aged 6 to 17 years from five Mediterranean nations: Italy, Spain, Portugal, Egypt, and Lebanon. Participants were recruited based on their voluntary consent to be included in a consumer database. Drawing from the recent literature with similar objectives in Mediterranean countries [22,23,24], a target of 400 participants was established for each Mediterranean country. Data collection was conducted through an electronic survey, resulting in a total of 2011 individuals being recruited. All procedures adhered to the Declaration of Helsinki (1989) by the World Medical Association, and each participant provided signed informed consent prior to joining the study.

### 2.2. Data Collection

Data on participants’ demographic characteristics and lifestyles were collected in 2019. For parents, information on sex, age, education level, and occupation was recorded, while for children/adolescents, gender, age, and anthropometric measurements were noted. The children’s/young people’s ages were divided into four categories: 6–8 years, 9–11 years, 12–14 years, and 15–17 years. Parental education was categorized into three levels: low (primary school), medium (secondary school), and high (tertiary education). Employment status was classified as unemployed or currently employed. Parents were asked about their children’s weight and height. The BMI of the children/adolescents was calculated based on their weight and height and classified according to the percentile ranges of the Centers for Disease Control and Prevention (CDC) growth charts for children and adolescents aged 2 to 19 years [25]. Participants were categorized as normal weight (BMI 5th–84th percentile), overweight (BMI 85th–94th percentile), and obese (BMI ≥ 95th percentile). Physical activity levels were measured using the International Physical Activity Questionnaire—Short Form (IPAQs), which collects information on physical activity levels (walking; moderate and vigorous intensity activities) over the past seven days, including the weekly frequency and daily duration of each activity, and classified as low, moderate, or high [26]. Participants were asked a series of questions concerning the eating habits of their children, including breakfast habits, location, frequency, company, and home-made vs. advertised food preference. Finally, screen time was divided into less than 2 h per day, 2–4 h per day, and more than 4 h per day.

### 2.3. Dietary Intake

Parents were asked to report what their children consumed over the past 24 h to document daily food intake. This included multiple response options categorized by eating occasion, as well as an open-ended option for any additional foods. To assess weekly food intake, questions regarding the frequency of consumption of key food groups were utilized.

### 2.4. Diet Quality

The Youth Healthy Eating Index (Y-HEI) is a modified adaptation of the Healthy Eating Index (HEI) specifically designed to assess dietary behaviors prevalent among children and adolescents [27]. Similarly to the HEI, the Y-HEI generates a total score ranging from 0 to 100, with higher values indicating superior diet quality. This index evaluates dietary intake based on 13 components, which are divided into two scoring categories: the first seven components are rated on a scale of 0 to 10, while the remaining six components are scored from 0 to 5. The initial three components assess the intake of whole grains, vegetables, and fruits. Dairy consumption is evaluated in the fourth component, wherein each serving of high-fat dairy products, such as whole milk and ice cream, is assigned half the score of lower-fat dairy alternatives. The fifth component quantifies protein intake by calculating the ratio of lean protein sources (i.e., poultry, fish, and tofu) to higher-fat meats (i.e., beef, pork, and lamb). The sixth component examines the consumption of snacks high in sugar or salt, whereas the seventh component assesses the intake of sugar-sweetened beverages, including regular soda, fruit punch, and sweetened iced tea. The remaining six components, each scored from 0 to 5, evaluate dietary habits and specific food choices. These include the use of multivitamins, margarine and butter intake, the consumption of fried foods outside the home, the presence of visible animal fat in meat (including skin), and behavioral aspects such as eating breakfast and dining with family. In this study, the components related to multivitamin use and visible fat intake were excluded, adjusting the maximum possible Y-HEI score to 90. This score has been demonstrated to be associated with better diet quality and country-specific national dietary recommendations in this study sample [28].

### 2.5. Sleep Duration

Sleep duration was assessed by asking participants how long, on average, their children sleep at night. The duration was categorized according to the National Sleep Foundation recommendations into adequate (reflecting 8–10 h) and inadequate (otherwise longer or shorter duration) [29].

### 2.6. Statistical Analysis

Categorical variables are shown as frequencies and percentages, and group differences are assessed using the Chi-square test. The Y-HEI variable was reported as mean and standard deviation (SD), with group differences evaluated using Student's *t*-test after the assessment of normality through the Kolmogorov–Smirnov test. Logistic regression analyses were conducted to compute the odds ratios (ORs) and 95% confidence intervals (CIs) for the relationships between tertiles and a 1 SD increase in Y-HEI scores and adequate sleep duration.

## 3. Results

A total of 77% of children and adolescents were deemed as having adequate sleep duration. The main demographic characteristics of the parents and children and adolescents participating in the study are presented in Table 1. Analyzing the distribution of the sample by background characteristics, it emerged that younger children with older parents were more likely to have an adequate sleep duration than their counterparts (OR = 0.55, 95% CI: 0.42, 0.72 and OR = 1.71, 95% CI: 1.35, 2.17, respectively). No other background variables resulted in affecting sleep duration (Table 1).

Table 2 shows the eating behaviors and physical activity of children/adolescents according to the adequacy of their sleep duration. Most variables explored (such as breakfast habit, eating outside of the home, eating with family, eating alone, eating advertised food, eating home-made food, screen time, and physical activity) were significantly associated with adequate sleep duration in the unadjusted models (Table 2); however, the multivariate analysis revealed that only the habits of always having breakfast and eating alone were independently related to adequate sleep duration (OR = 2.68, 95% CI: 2.03, 3.54 and OR = 0.62, 95% CI: 0.42, 0.91, respectively).

The eating habits of children and adolescents according to sleep duration adequacy are presented in Table 3. A positive association was found for most variables regarding the consumption of more than one serving of fruit, cereals, dairy, meat, fish, legumes, and whole grains. However, the associations for some foods were significant only when considering moderate consumption (i.e., 1–2 portions/d).

The mean values of the Y-EHI scores in children and adolescents with adequate vs. inadequate sleep duration were 52.2 ± 11.5 and 49.3 ± 11.7, respectively (*p* < 0.001). Adjusting for all the background, eating, and lifestyle variables previously found significantly related to the outcome, children and adolescents in the highest tertile of Y-EHI scores were 63% more likely to have an adequate sleep duration (OR = 1.63, 95% CI: 1.24, 2.16, Table 4); interestingly, the diet quality score was linearly associated with the probability to have adequate sleep duration (for a 1 SD increase, OR = 1.23, 95% CI: 1.10, 1.38, Table 4).

## 4. Discussion

This research aimed to explore the lifestyle and dietary factors associated with adequate sleep duration among younger populations in the Mediterranean region. Specifically, targeting children and adolescents from five countries, this study examined dietary habits and other lifestyle habits across these diverse areas. By doing so, it sought to provide a comprehensive understanding of the factors influencing sleep duration within different cultural and geographical contexts of the Mediterranean basin.

In the present study, the association between better sleep adequacy and lifestyle/eating factors of children and adolescents was demonstrated. The consumption of individual food groups, as well as healthy dietary patterns and sleep duration, has been widely reported in the literature [30]. There are several studies that have highlighted a potential relation between healthier dietary patterns or individual food groups and sleep quality, and some general characteristics were common to all dietary patterns examined in a systematic literature review, including a high intake of plant-based foods, such as fruits and vegetables, whole grains and legumes, olive oil and seafood as the main sources of fats, and a low consumption of processed foods and those rich in free sugars, for example, sugary beverages [31]. Diets and lifestyles with such features have been hypothesized to positively influence various aspects of sleep, including sleep duration, quality, and overall sleep efficiency [32].

In this study, a positive association between the consumption of more than one serving of fruit and a better sleep duration was reported. This suggests that fruit, as part of a balanced diet, may have a beneficial impact on sleep quality and duration [33]. Fruits are rich in vitamins (such as vitamins B and C) and minerals (such as potassium and zinc), which play a role in sleep quality, preserve memory during aging, and improve cerebral and cognitive functions, and when deficiencies were corrected, an improvement in both sleep duration and overall sleep quality was noted [34]. Fruit is also rich in tryptophan, melatonin, and serotonin, which are important for improving sleep quality: in adults, after consuming foods rich in tryptophan, individuals often experience a longer time to fall asleep, better overall performance throughout the day, and a significant increase in the total duration of sleep [35]. Additionally, certain fruits contain natural sugars and complex carbohydrates that can help regulate blood sugar levels and support stable energy throughout the day, potentially leading to improved sleep at night. The presence of antioxidants in fruits may also help reduce oxidative stress and inflammation, further promoting healthy sleep patterns [3,36].

Regarding primary animal protein sources, it was observed that individuals who consumed higher amounts of more than three portions/d of meat, fish, and dairy demonstrated a positive association with more adequate sleep duration. This finding highlights the potential role of high-quality protein-rich foods in supporting better sleep patterns. Meat and fish provide essential amino acids, such as tryptophan, which is a precursor for serotonin and melatonin, which are key regulators of sleep–wake cycles [37]. A diet abundant in fish and seafood has also been shown to promote better sleep, particularly due to the presence of omega-3 fatty acids [38]. Several studies suggest the potential benefits of seafood and omega-3 fatty acids for brain health and sleep quality. For example, docosahexaenoic acid (DHA) is a key structural component of neuron membranes, supporting their stability and homeostasis, while also influencing neurotransmission pathways such as serotoninergic, noradrenergic, and dopaminergic pathways [39]. Also, Omega-3 fatty acids are believed to impact brain health through mechanisms such as the neuroendocrine modulation of neurotransmission and the synthesis of neurotrophic factors [40]. Dairy, such as milk, yogurt, and cheese, is rich in nutrients like calcium, magnesium, and vitamin D, which are known to play a role in regulating sleep [41]. Calcium, in particular, helps the brain use tryptophan to produce melatonin, a hormone that regulates sleep patterns. Additionally, dairy products contain a fair amount of protein, which can help maintain stable blood sugar levels and prevent nighttime awakenings [42]. These results suggest that incorporating a variety of nutrient-dense protein sources into one’s diet may contribute to enhancing both the quality and duration of sleep [43].

Another significant connection has been observed between the quality of carbohydrates and plant protein consumed (such as whole grains and legumes high in fiber content) and an overall adequate sleep duration [44]. Whole grains, such as oats, brown rice, and whole wheat, are rich in fiber, vitamins, and minerals that contribute to overall health [45]. Legumes, in addition to being a good source of plant-based protein, are rich in fiber and complex carbohydrates, which may further support stable energy levels and promote restful sleep [46]. The high fiber content in whole grains and legumes can help regulate blood sugar levels, preventing spikes and crashes that may interfere with sleep [47]. The glycemic index, along with the timing and frequency of meals, is influenced not only by the quantity of carbohydrates consumed but also by their nutritional quality [48]. Furthermore, whole grains and legumes are a good source of complex carbohydrates, which promote the production of serotonin, a neurotransmitter that helps regulate mood and sleep. Such data regarding food intake are supported by other studies that have shown that insufficient macronutrient intake, excessive calorie consumption, and eating meals late in the evening contribute to a decline in sleep quality and may increase the risk of developing insomnia [49].

Examining eating habits revealed notable variations in sleep duration, particularly when comparing individuals based on their breakfast consumption patterns; children and adolescents who consistently ate breakfast every day were more likely to have adequate sleep duration compared to those who skipped this meal. This trend suggests a potential link between regular breakfast consumption and healthier sleep routines, emphasizing the interconnected nature of dietary habits and overall well-being [50]. Skipping breakfast and having inconsistent meal times may also contribute to instability in blood sugar levels, potentially leading to difficulty falling and staying asleep [51]. These findings highlight the importance of understanding how daily routines, such as meal timing and frequency, influence other lifestyle factors, including sleep quality and duration [52].

Similar findings were observed regarding eating with family and consuming homemade food. Specifically, participants who regularly ate meals with their family or preferred homemade food displayed trends similar to those seen with breakfast consumption, showing positive effects on sleep duration and quality. These results highlight how the context in which meals are consumed and food choices can play crucial roles in promoting a balanced lifestyle, enhancing both dietary habits and overall well-being [53]. Consistent with these findings, it was observed that individuals who avoided eating outside of the home and never eating alone tended to have an adequate sleep duration. Regarding food choices, it was found that participants who preferred non-advertised foods exhibited adequate sleep duration and quality compared to those who favored advertised products. This finding suggests that a balanced diet, excluding junk food and highly processed foods often promoted through advertising campaigns, is associated with improved sleep habits [54]. Other studies have explored the association between lifestyle habits and sleep, demonstrating that the excessive intake of pasta, sweets, and sugary beverages, all foods that are widely promoted by advertisements or that are commonly eaten outside the home, coupled with skipping breakfast and irregular eating patterns, is linked to poor sleep quality [55].

This study also found that some background demographic characteristics of parents and children might also play a role in the adequacy of sleep duration. A higher percentage of adolescents have inadequate sleep duration compared to children. This result may be attributed to the fact that children are more frequently monitored and encouraged by their parents to adhere to an earlier bedtime, whereas adolescents, at this stage, begin to establish their own routines, which are less influenced by parental guidance [56]. Moreover, among the most commonly reported barriers to adequate sleep in adolescents are the early start times of school activities, the widespread and often uncontrolled use of technological devices, and the profound physiological changes occurring during puberty [57]. These include alterations in circadian rhythms, which tend to shift the biological clock toward an evening chronotype, and changes in sleep homeostasis, which affect the ability to fall asleep and maintain high-quality sleep [58]. These factors, interacting with one another, contribute to reducing both the duration and the effectiveness of nocturnal rest, with significant implications for the psychological and physical well-being as well as the cognitive performance of adolescents [59]. Conversely, regarding parents, it was found that children with parents over 45 years of age have a higher likelihood of having more adequate sleep compared to those having younger parents. It may be possible that more experience, which tends to improve with age, might help manage children’s behaviors and healthy habits, including sleep hygiene [60].

To the best of our knowledge, this research offers a valuable opportunity to analyze dietary and lifestyle factors linked to sufficient sleep duration among children and adolescents in five Mediterranean countries. The strengths of this study include the large sample size, a standardized methodology used to collect data, and harmonized results for all countries involved. Nonetheless, the findings must be interpreted with caution, given certain limitations. The cross-sectional nature of this study prevents establishing causal relationships. Additionally, potential reporting bias may arise from the reliance on parent-completed questionnaires regarding their children’s dietary patterns and eating behaviors. Also, data on children’s anthropometric measures were reported by the parents, which potentially raises the possibility of recall bias. Another limitation is related to the self-reporting survey method, with no possibility of obtaining clarification of doubts during the completion of the questionnaires (i.e., from a dietician). Finally, some variables used to calculate the Y-HEI were missing, hence potentially diminishing the reliability to assess the diet quality of this instrument in its full capacity.

## 5. Conclusions

In conclusion, the adequacy of sleep in children and adolescents in the Mediterranean region is influenced by various lifestyle and dietary factors. Habits such as eating breakfast, dining with family, engaging in physical activity, and choosing healthy foods like fruits, whole grains, fish, and legumes are linked to better sleep adequacy. Nonetheless, a more comprehensive evaluation of sleep quality, including various sleep features in addition to sleep duration, is highly warranted. This highlights the importance of balanced lifestyles and healthy diets for overall health. This study calls for an integrated approach, combining dietary education and lifestyle promotion, with collaboration among parents, educators, and healthcare professionals. Creating a supportive environment with practical resources and strategies can foster healthier choices, improving both dietary habits and physical activity, and ultimately enhancing children’s overall well-being and health outcomes.

## Figures and Tables

**Table 1:** Demographic characteristics of parents and children and adolescents participating in the study according to the sleep duration adequacy (n = 2011).

	Sleep Duration			Sleep Duration	
	Inadequate	Adequate	*p*-Value	OR (95% CI)	OR (95% CI) *
**Age groups, (n, %)**			0.019		
Children (6–11 y)	222 (47.3)	825 (53.5)		1	1
Adolescents (12–17 y)	247 (52.7)	717 (46.5)		0.78 (0.64, 0.96)	0.55 (0.42, 0.72)
**Sex, (n, %)**			0.141		
Male	246 (52.5)	749 (48.6)		1	1
Female	223 (47.5)	793 (51.4)		1.17 (0.95, 1.44)	1.23 (0.95, 1.60)
**Weight status, (n, %)**			0.620		
Normal weight	210 (66.5)	877 (69.3)		1	1
Overweight	57 (18.0)	206 (16.3)		0.87 (0.62, 1.20)	0.86 (0.60, 1.23)
Obese	49 (15.5)	183 (14.5)		0.89 (0.63, 1.27)	0.85 (0.58, 1.24)
**Parents’ age, (n, %)**			<0.001		
<44 y	133 (28.4)	290 (18.8)		1	1
≥45 y	336 (71.6)	1252 (81.2)		1.71 (1.35, 2.17)	2.07 (1.48, 2.88)
**Parents’ occupational level, (n, %)**			0.099		
Unemployed	343 (74.6)	1190 (78.2)		1	1
Current working	117 (25.4)	331 (21.8)		0.82 (0.64, 1.04)	1.21 (0.86, 1.71)
**Parents’ educational level, (n, %)**			0.029		
Low	31 (7.0)	60 (4.0)		1	1
Medium	163 (37.0)	587 (39.3)		1.86 (1.17, 2.97)	1.81 (0.97, 3.38)
High	247 (56.0)	846 (56.7)		1.77 (1.12, 2.79)	1.80 (0.96, 3.37)
**Area of living, (n, %)**			0.696		
Urban	377 (80.4)	1252 (81.2)		1	1
Rural	92 (19.6)	290 (18.8)		0.95 (0.73, 1.23)	0.93 (0.66, 1.31)

* analyses were adjusted for all variables presented in the table.

**Table 2:** Eating behaviors and physical activity of children and adolescents according to sleep duration adequacy (n = 2011).

	Sleep Duration			Sleep Duration	
	Inadequate	Adequate	*p*-Value	OR (95% CI)	OR (95% CI) *
**Breakfast habit, (n, %)**			<0.001		
Never/seldom	106 (22.6)	171 (11.1)		1	1
Often	103 (22.0)	245 (15.9)		1.47 (1.06, 2.06)	1.34 (0.95, 1.89)
Always	260 (55.4)	1126 (73.0)		2.68 (2.03, 3.54)	2.23 (1.62, 3.08)
**Eating outside of the home, (n, %)**			0.017		
Never	199 (42.4)	733 (47.5)		1	1
1 time	212 (45.2)	679 (44.0)		0.87 (0.70, 1.08)	1.14 (0.88, 1.47)
2 or more times	58 (12.4)	130 (8.4)		0.61 (0.43, 0.86)	0.74 (0.50, 1.09)
**Eating with family, (n, %)**			<0.001		
Seldom	17 (3.6)	24 (1.6)		1	1
Often	179 (38.2)	422 (27.4)		1.67 (0.88, 3.18)	1.20 (0.60, 2.38)
Daily	273 (58.2)	1096 (71.1)		2.84 (1.51, 5.37)	1.40 (0.71, 2.79)
**Eating alone, (n, %)**			<0.001		
Never/seldom	239 (51.0)	1008 (65.4)		1	1
Often	178 (38.0)	419 (27.2)		0.56 (0.45, 0.70)	0.72 (0.53, 0.97)
Daily	52 (11.1)	115 (7.5)		0.52 (0.37, 0.75)	0.62 (0.42, 0.91)
**Eating at school, (n, %)**			0.722		
Never/seldom	201 (42.9)	630 (40.9)		1	1
Often	143 (30.5)	479 (31.1)		1.07 (0.84, 1.37)	1.33 (1.01, 1.74)
Almost daily	125 (26.7)	433 (28.1)		1.11 (0.86, 1.43)	1.06 (0.81, 1.39)
**Eating advertised foods, (n, %)**			0.005		
No	214 (45.6)	817 (53.0)		1	1
Yes	255 (54.4)	725 (47.0)		0.74 (0.61, 0.92)	0.93 (0.73, 1.19)
**Eating home-made foods, (n, %)**			0.004		
Seldom	69 (14.7)	183 (11.9)		1	1
Often	228 (48.6)	663 (43.0)		1.10 (0.80, 1.50)	1.26 (0.89, 1.79)
Almost daily	172 (36.7)	696 (45.1)		1.53 (1.10, 2.11)	1.23 (0.87, 1.72)
**Screen time, (n, %)**			0.080		
<2 h/day	255 (54.4)	876 (56.8)		1	1
2–4 h/day	166 (35.4)	557 (36.1)		0.98 (0.78, 1.22)	1.70 (1.27, 2.27)
>4 h/day	48 (10.2)	109 (7.1)		0.66 (0.46, 0.95)	1.26 (0.97, 1.63)
**Physical activity level, (n, %)**			<0.001		
Low	274 (58.4)	743 (48.2)		1	1
Medium	79 (16.8)	382 (24.8)		1.78 (1.35, 2.36)	1.05 (0.83, 1.33)
High	116 (24.7)	417 (27.0)		1.33 (1.03, 1.70)	0.86 (0.58, 1.27)

* analyses were adjusted for all variables presented in the table.

**Table 3:** Food group consumption of children and adolescents according to the sleep duration adequacy (n = 2011).

	Sleep Duration			Sleep Duration	
	Inadequate	Adequate	*p*-Value	Univariate OR (95% CI)	Multivariate OR (95% CI) *
**Vegetables, n (%)**			0.019		
Never	35 (7.5)	90 (5.8)		1	1
1–2 portions/d	365 (77.8)	1287 (83.5)		1.37 (0.91, 2.06)	0.97 (0.61, 1.53)
≥3 portions/d	69 (14.7)	165 (10.7)		0.93 (0.57, 1.50)	0.66 (0.38, 1.16)
**Fruit, n (%)**			<0.001		
Never	40 (8.5)	53 (3.4)		1	1
1–2 portions/d	340 (72.5)	1208 (78.3)		2.68 (1.75, 4.11)	2.13 (1.33, 3.42)
≥3 portions/d	89 (19.0)	281 (18.2)		2.38 (1.48, 3.83)	2.07 (1.20, 3.59)
**Cereals, n (%)**			0.005		
Never	25 (5.3)	81 (5.3)		1	1
1–2 portions/d	378 (80.6)	1323 (85.8)		1.08 (0.68, 1.72)	0.88 (0.54, 1.43)
≥3 portions/d	66 (14.1)	138 (8.9)		0.65 (0.38, 1.10)	0.59 (0.33, 1.05)
**Dairy, n (%)**			<0.001		
Never	128 (27.3)	276 (17.9)		1	1
1–2 portions/d	230 (49.0)	931 (60.4)		1.88 (1.46, 2.42)	1.61 (1.24, 2.11)
≥3 portions/d	111 (23.7)	335 (21.7)		1.40 (1.04, 1.89)	1.24 (0.90, 1.72)
**Meat, n (%)**			<0.001		
Never	50 (10.7)	93 (6.0)		1	1
1–2 portions/w	256 (54.6)	785 (50.9)		1.65 (1.14, 2.39)	1.21 (0.80, 1.84)
≥3 portions/w	163 (34.8)	664 (43.1)		2.19 (1.49, 3.22)	1.59 (1.02, 2.47)
**Legumes, n (%)**			0.004		
Never	37 (7.9)	64 (4.2)		1	1
1–2 portions/w	310 (66.1)	1085 (70.4)		2.02 (1.32, 3.09)	1.61 (1.02, 2.54)
≥3 portions/w	122 (26.0)	393 (25.5)		1.86 (1.18, 2.93)	1.40 (0.85, 2.31)
**Fish, n (%)**			<0.001		
Never	93 (19.8)	192 (12.5)		1	1
1–2 portions/w	307 (65.5)	1063 (68.9)		1.68 (1.27, 2.22)	1.24 (0.90, 1.72)
≥3 portions/w	69 (14.7)	287 (18.6)		2.01 (1.40, 2.89)	1.62 (1.05, 2.50)
**Whole grains, n (%)**			<0.001		
Never	166 (35.4)	401 (26.0)		1	1
1–2 portions/w	192 (40.9)	617 (40.0)		1.33 (1.04, 1.70)	1.24 (0.97, 1.60)
≥3 portions/w	111 (23.7)	524 (34.0)		1.95 (1.49, 2.57)	1.81 (1.35, 2.41)
**Sweets, n (%)**			0.334		
Never	31 (6.6)	129 (8.4)		1	1
1–2 portions/w	224 (47.8)	691 (44.8)		0.74 (0.49, 1.13)	0.66 (0.42, 1.02)
≥3 portions/w	214 (45.6)	722 (46.8)		0.81 (0.53, 1.23)	0.76 (0.48, 1.19)

* analyses were adjusted for all variables presented in the table. Abbreviations: d (day); w (week).

**Table 4:** Association between diet quality of children and adolescents and adequate sleep duration (n = 2011).

	Y-HEI, OR (95% CI)			
	T1	T2	T3	1 SD Increase
**Adequate sleep duration**				
Model 1	1	1.40 (1.10, 1.78)	2.02 (1.55, 2.63)	1.36 (1.22, 1.51)
Model 2	1	1.37 (1.07, 1.75)	1.90 (1.46, 2.48)	1.32 (1.19, 1.47)
Model 3	1	1.21 (0.94, 1.55)	1.63 (1.24, 2.16)	1.23 (1.10, 1.38)

Model 1, unadjusted; Model 2, adjusted for age groups and parents’ age; Model 3, adjusted for breakfast habit, eating outside of the home, and eating at school.

## Data Availability

All data are available upon reasonable request. The data are not available publicly due to being a part of an ongoing study.
